# Comprehensive Analysis of Codon Usage on Porcine Astrovirus

**DOI:** 10.3390/v12090991

**Published:** 2020-09-06

**Authors:** Huiguang Wu, Zhengyu Bao, Chunxiao Mou, Zhenhai Chen, Jingwen Zhao

**Affiliations:** 1 College of Veterinary Medicine, Yangzhou University, Yangzhou 225009, China; huiguang-wu@yzu.edu.cn (H.W.); bzy199703@163.com (Z.B.); ytqxmcx@163.com (C.M.); 2 Institute of Comparative Medicine, Yangzhou University, Yangzhou 225009, China; 3 Jiangsu Co-Innovation Center for Prevention and Control of Important Animal Infectious Diseases and Zoonoses, Yangzhou University, Yangzhou 225009, China; 4 Joint International Research Laboratory of Agriculture and Agri-Product Safety, The Ministry of Education of China, Yangzhou University, Yangzhou 225009, China; 5 College of Animal Science and Technology, Yangzhou University, Yangzhou 225009, China

**Keywords:** porcine astrovirus (PAstV), phylogenetic analysis, codon usage pattern, natural selection, mutation pressure, host adaptability

## Abstract

Porcine astrovirus (PAstV), associated with mild diarrhea and neurological disease, is transmitted in pig farms worldwide. The purpose of this study is to elucidate the main factors affecting codon usage to PAstVs. Phylogenetic analysis showed that the subtype PAstV-5 sat at the bottom of phylogenetic tree, followed by PAstV-3, PAstV-1, PAstV-2, and PAstV-4, indicating that the five existing subtypes (PAstV1-PAstV5) may be formed by multiple differentiations of PAstV ancestors. A codon usage bias was found in the PAstVs-2,3,4,5 from the analyses of effective number of codons (ENC) and relative synonymous codon usage (RSCU). Nucleotides A/U are more frequently used than nucleotides C/G in the genome CDSs of the PAstVs-3,4,5. Codon usage patterns of PAstV-5 are dominated by mutation pressure and natural selection, while natural selection is the main evolutionary force that affects the codon usage pattern of PAstVs-2,3,4. The analyses of codon adaptation index (CAI), relative codon deoptimization index (RCDI), and similarity index (SiD) showed the codon usage similarities between the PAstV and animals might contribute to the broad host range and the cross-species transmission of astrovirus. Our results provide insight into understanding the PAstV evolution and codon usage patterns.

## 1. Introduction

Porcine astroviruses (PAstVs), comprising five distinct lineages (PAstV1-PAstV5), are highly prevalent in both diarrheic and clinically healthy pigs [1,2,3]. PAstV-3 is found in tissues from the central nervous system of piglets and sows with encephalomyelitis and neural necrosis [4,5]. Infection of piglets with PAstV-1 could cause mild diarrhea, growth retardation, and damage to the villi of the small intestinal mucosa [6]. PAstV-4 was detected in the nasal swabs [7] and the feces of pigs [8,9]. Co-infection of individual pigs with several lineages of PAstVs has also been observed [9]. Both PAstV-2 and PAstV-5 have been identified in the brains of newborn piglets with congenital tremors [10]. PAstV-2 and PAstV4 were simultaneously detected in the blood samples of apparently healthy domestic pigs, while the coexistence of PAstV-2, PAstV-4, and PAstV-5 has been observed in porcine fecal samples collected from the same farms [9,11].

PAstV is a non-enveloped, single-stranded, positive-sense RNA virus belonging to the genus *Mamastrovirus* within the family *Astroviridae*. The PAstV genome contains three overlapping open reading frames (ORFs) encoding nonstructural proteins (ORF1a), RNA-dependent RNA polymerase (ORF1b), and viral capsid proteins (ORF2) [9]. As a typical genome organization of the genus *Mamastrovirus*, the highly conserved ribosomal frameshifting signal (5′-AAAAAAC-3′) is present precisely in the overlapping region of ORF1a and ORF1b of PAstV, which is essential for the translation of RNA polymerase [12]. The expression of ORF1b is mediated through programmed ribosomal frameshifting into ORF1b [13]. The PAstV capsid protein controls the initial phases of virus infection, including virus attachment, endocytosis, and genome release into the host cells [14].

The codon usage pattern reveals the basic features of molecular evolution [15]. Except for the codons of methionine (Met) and tryptophan (Trp), others encoding the same amino acid are termed synonymous codons. The codon usage of a species is not random, because some synonymous codons are more frequently used than others. This phenomenon of non-randomness in synonymous codon usage is called codon bias [16]. The viral codon usage may be influenced by the host, because the viral replication needs its host machinery. Although previous analysis revealed that host-affected nucleic acid composition and codon usage were the drivers of *Astroviridae* evolution [17], knowledge of codon usage patterns of PAstV is still very limited. Consequently, we employed a number of methods to investigate the evolutionary processes of PAstV by codon usage pattern analysis in this study.

## 2. Materials and Methods

### 2.1. Sequence Data

In this study, 69 complete coding sequences of PAstVs genomes were retrieved from the National Center for Biotechnology Information (NCBI) nucleotide database (http://www.ncbi.nlm.nih.gov) recorded up to January 2020. Considering the limited number of sequences, PAstV-1 was excluded from the study of codon usage. To investigate overall codon usage bias in the PAstVs genomes, stop codons of ORF1a and ORF1b were removed, and ORFs were concatenated in the following order: ORF1a-ORF1b-ORF2. Detailed information concerning PAstV genomes, including the accession number, strain name, geographical distribution of isolated strains, and the isolation year, is listed in Table S1.

### 2.2. Phylogenetic Analysis

The coding DNA sequences (CDSs) of PAstV genomes were aligned using MACSE [18] (version 2.03). The best-fit model of nucleotide substitution was identified using jModelTest2 [19] (version 2.1.10) according to corrected Akaike Information Criterion (AICc). The general time-reversible (GTR) with a gamma-distributed evolutionary rates (G) and invariant sites (I) (GTR + G + I) were selected as the best-fit model of nucleotide substitution based on the criteria mentioned above. The maximum likelihood (ML) phylogenetic tree was inferred using RAxML [20] (version 8.2.12) with model GTR + G + I. The node support was assessed by performing 10,000 bootstrap iterations. The Bayesian inference (BI) phylogeny was reconstructed using Mrbayes [21] (version 3.2.7a) with the model GTR + G + I. The Markov Chain Monte Carlo (MCMC) search was conducted for 10,000,000 generations, and the posterior probabilities were estimated for each node. The first 25% of sampled trees were discarded as burn-in. The phylogenetic trees were viewed using Figtree [22] (version 1.4.4).

### 2.3. Nucleotide Composition

Considering the limited number of sequences, PAstV-1 was excluded, while the remaining four subtypes of PAstVs (PAstV-2 to PAstV-5) were investigated. The frequencies of mononucleotides (A, C, U, and G), GC, GC1s (GC content at the first codon positions), GC2s (GC content at the second codon positions), GC12s (the mean value of GC1s and GC2s), and GC3s (GC content at the third codon positions) were calculated using the seqinr package (version 3.6–1) [23] of R (version 3.6.2) [24]. The frequencies of mononucleotides at the third synonymous codon position (A3s, C3s, U3s, and G3s) were calculated using CodonW software (version 1.4.2) developed by J. Peden (http://codonw.sourceforge.net/).

To evaluate the possible effects of relative dinucleotide abundances on codon usage of PAstV, the odds ratio of observed to expected dinucleotide (O/E) frequency was computed according to the equation below [25]:

(1) $$P_{xy}=\frac{f_{xy}}{f_{x}f_{y}}$$

where *f_x_*, *f_y_*, *f_x_f_y_*, and *f_xy_* represent the frequency of nucleotide X, nucleotide Y, the expected frequency of dinucleotide XY, and the frequency of dinucleotide XY, respectively.

### 2.4. Effective Number of Codons (ENC)

The effective number of codons is designed to quantify how far the codon usage of a gene departs from equal usage of synonymous codons, regardless of the gene lengths and the number of amino acids [26]. The value of ENC ranges from 20 (if only one synonymous codon is exclusively used for the corresponding amino acid) to 61 (if all of the synonymous codons are used with no preference) [26,27]. The smaller the ENC value of a gene is, the stronger the extent of codon preference of this gene. ENC values were calculated using the following Equation [26]:

(2) $$ENC=2+\frac{9}{F_{2}}+\frac{1}{F_{3}}+\frac{5}{F_{4}}+\frac{3}{F_{6}}$$

where *F_i_* (*i* = 2, 3, 4, 6) represents the mean of *F_i_* values for the *i*-fold degenerate amino acids. *F_i_* can be calculated using the equation below [26]:

(3) $$F_{i}=\frac{n\sum_{j=1}^{i}{\left(\frac{n_{j}}{n}\right)}^{2}-1}{n-1}$$

where *n* represents the total number of observed codons for that amino acid; and *n_j_* represents the total observed number of the *j*th codon for that amino acid. The ENC values for PAstVs CDSs were calculated using the cordon package (version 1.4.0) [28] of R (version 3.6.2) [24].

### 2.5. Relative Synonymous Codon Usage (RSCU)

To measure the non-uniform usage of synonymous codons in a coding sequence, RSCU is defined as the ratio of observed to expected codon frequency under equal codon usage without being affected by the amino acid compositions or the CDS sizes of different gene samples [16]. Synonymous codons with RSCU values <1.0, =1.0, and >1.0 represent negative codon usage bias, no bias, and positive codon usage bias, respectively. Furthermore, synonymous codons with RSCU values >1.6 and <0.6 were regarded as “overrepresented” and “underrepresented” codons, respectively [29]. The RSCU was calculated as:

(4) $$RSCU=\frac{g_{ij}}{\sum_{j}^{n_{i}}g_{ij}}n_{i}$$

where *g_ij_* represents the observed number of the *i*th codon for the *j*th amino acid, which is encoded by *n_i_* synonymous codons [30]. The RSCU index was calculated for each sequence using the seqinr package (version 3.6–1) [23] of R (version 3.6.2) [24].

### 2.6. Principal Component Analysis (PCA)

Principal component analysis (PCA) is a multivariate statistical method that reduces data dimensionality by performing a covariance analysis between factors [31]. To investigate the dominant patterns and variations in the codon usage of PAstVs CDSs, we performed a PCA with the RSCU values of the PAstVs genome. For transforming the RSCU values into uncorrelated variables, the RSCU value of each PAstV sequence was distributed into a 59-dimensional vector corresponding to the 59 synonymous codons by excluding AUG, UGG, and three terminal codons. A matrix comprising 59 RSCU values of each sequence was built for the PCA and transformed into several major axes. PCA was performed on the obtained RSCU dataset by using the factoextra package (version 1.0.6) [32] of R (version 3.6.2) [24].

### 2.7. ENC-Plot Analysis

In the ENC-plot analysis, the projection of ENC-values versus GC3s is commonly used to explore factors influencing the codon usage patterns, e.g., selection [26]. In an ENC plot, the observed and expected ENC values are compared to determine the influence of structuring synonymous codon usage bias. The expected ENC values for all of the GC3 compositions, ranging from 0 to 1, were calculated using the following equation:

(5) $$ENC_{expected}=2+s+\frac{29}{s^{2}+{\left(1-s\right)}^{2}}$$

where *s* is the frequency of G + C at the third codon position of synonymous codons. An expected curve was generated using the expected ENC values. In the ENC-GC3s plot, if observed ENC values fell on the curve of expected ENC values, it meant that mutation was the main force acting on third-position bases of codons, whereas if observed ENC values fell considerably below the expected curve, it meant that selection was the main force driving codon usage bias.

### 2.8. Neutrality Plot Analysis

A neutrality plot was used to identify the effects of natural selection and mutation pressure on the codon usage patterns [33]. The obtained GC3 and GC12 values (means of GC1 and GC2) of the synonymous codons were plotted on the horizontal and vertical axes, respectively, to produce a scatter diagram for the neutrality plot. The regression line was plotted between the GC3-variable and the GC12-variable. The slope (regression coefficient) of the regression line is regarded as the mutation-selection equilibrium coefficient [33]. If all of the points are distributed along the diagonal (slope = 1) and the correlation between GC3-variable and GC12-variable is statistically significant, this indicates that mutation is the main force shaping the codon usage. Alternatively, if the regression curve is parallel or tilted toward the horizontal axis (close to zero slope), selection is considered as the dominant factor. The regression analysis, which estimates the linear relationship between GC3-variable and GC12-variable, was performed by using R (version 3.6.2) [24].

### 2.9. Parity Rule 2 (PR2) Analysis

Parity rule 2 (PR2) plot analysis is another method used to investigate the influence of mutation and selection on codon usage. In the PR2 plot, AT bias [A3/(A3 + T3)] and GC bias [G3/(G3 + C3)] were chosen as the ordinate and abscissa, respectively. The center of the plot, i.e., A = U and G = C (PR2), defined as coordinates of the origin (0.5, 0.5), indicates no bias between the influences of mutation pressure and natural selection [34,35].

### 2.10. Codon Adaptation Index (CAI) Analysis

Codon adaptation index (CAI) is a quantitative measure for assessing the codon usage similarities between viral genes and their hosts [36]. The values of CAI range from 0 to 1. The virus sequences with higher CAI values are considered to be preferred over those with lower CAI values. The CAI analysis of the PAstV coding sequences was performed with CAIcal [36]. The reference datasets of synonymous codon usage patterns of chicken (*Gallus gallus*), duck (*Anas platyrhynchos platyrhynchos*), human (*Homo sapiens*), dog (*Canis lupus familiaris*), horse (*Equus caballus*), mouse (*Mus musculus*), pig (*Sus scrofa*), cat (*Felis catus*), cattle (*Bos taurus*), goat (*Capra hircus*) and sheep (*Ovis aries*) were downloaded from the Codon and Codon Pair Usage Tables (CoCoPUTs) database updated in January 2020 [37].

### 2.11. Relative Codon Deoptimization Index (RCDI)

RCDI is used to estimate the codon usage deoptimization trend of a virus to its host [38]. In a virus, the RCDI value of 1 indicates the virus has complete host-adapted codon usage pattern, while a value of RCDI higher than 1 indicates low adaptability to a host [39]. RCDI values of PAstV sequences were calculated using CAIcal [36].

### 2.12. Similarity Index (SiD) Analysis

To measure the influence of the codon usage patterns of the host on codon usage bias of PAstVs CDSs, a SiD analysis was performed. SiD is calculated using the following equation:

(6) $$R\left(A,B\right)=\frac{\sum_{i=1}^{59}a_{i}\times b_{i}}{\sqrt{\sum_{i=1}^{59}a_{i}^{2}\times \sum_{i=1}^{59}b_{i}^{2}}}$$

(7) $$D\left(A,B\right)=\frac{1-R\left(A,B\right)}{2}$$

where *R*(*A*,*B*) is defined as the cosine of the angle included between the *A* and *B* spatial vectors; *a_i_* means the RSCU value of 59 synonymous codons of the PAstV coding sequence; *b_i_* is the RSCU value of the same codon in the host; and *D(A,B)* represents the potential effect of the overall codon usage of the host on that of PAstV [40]. A high value of SiD indicates that the host has dominant effects on the codon usages of the virus.

### 2.13. Statistical Analysis

A non-parametric Kruskal–Wallis test was separately used to determine any significant differences between the values of CAI, RCDI and SiD of the four PAstV subtypes. The *p*-values for Dunn’s multiple comparisons were adjusted with the Benjamini–Hochberg method. The level of significance was set at *p* < 0.05. The statistical analysis was performed with the package dunn.test (version 1.3.5) [41] of R (version 3.6.2) [24].

## 3. Results

### 3.1. PAstV-5 Subtype Has the Basal Position of Phylogenetic Tree

**Figure 1:**
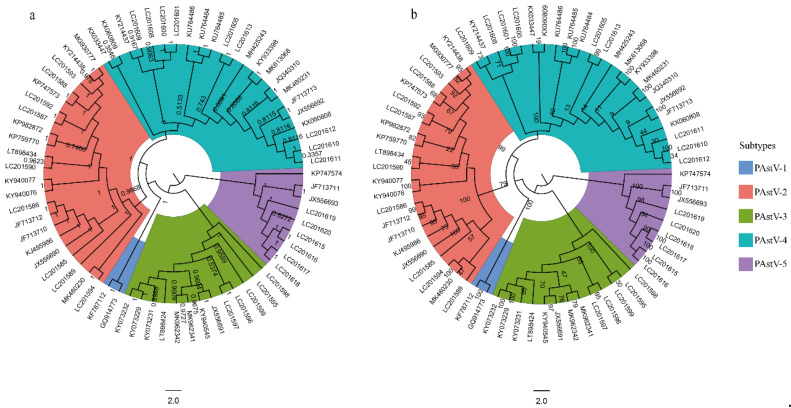
Phylogenetic trees of 69 complete genomes of PAstV. (**a**) BI tree of the PAstV genomes reconstructed by MrBayes. Posterior probability values are shown at each node. (**b**) ML tree of the PAstV genomes reconstructed by RAxML. Bootstrap support values are indicated on the tree as a percentage of 10,000 replicates. The colored circular sectors indicate the five subtypes of PAstV.

To explore the phylogeny among the five subtypes of PAstV strains, we constructed the phylogenetic trees using ML and BI methods. Results showed the topologies of the BI and ML trees were identical (Figure 1). The subtype PAstV-5 was a basal clade in the BI and ML trees, followed by PAstV-3, PAstV-1, PAstV-2, and PAstV-4, suggesting the ancestors of PAstV may have undergone multiple differentiations before forming the five existing subtypes.

### 3.2. A and U Nucleotides Were More Frequent than C and G in the PAstV Coding Sequences

Nucleotide A was the most represented in the genome CDSs of PAstV-2 (0.268 ± 0.004) and PAstV-4 (0.301 ± 0.003) (Table 1 and Table S2). Content of nucleotide U was highest in the genome CDSs of PAstV-3 (0.279 ± 0.002) and PAstV-5 (0.269 ± 0.004). Further analysis of third-position wobble nucleotides revealed that U3 was the highest in the genome of PAstV-3 (0.433 ± 0.01), PAstV-4 (0.399 ± 0.011), and PAstV-5 (0.421 ± 0.009), whereas C3 was the most dominant in PAstV-2 (0.371 ± 0.011). The GC content in the coding sequence of PAstV was not uniformly distributed. In all of the four PAstV subtypes, the GC1 content was the highest compared to the contents of GC2, GC12, and GC3. The frequency values of GC3s of PAstV-2, PAstV-3, and PAstV-5 strains were higher than those of GC2s. The PAstV-2 has the highest ENC value (56.265 ± 0.602), followed by PAstV-5 (53.647 ± 0.316), PAstV-3 (53.059 ± 0.656), and PAstV-4 (52.007 ± 0.678). GC3 content was highest in PAstV-2 (0.534 ± 0.012), followed by PAstV-5 (0.467 ± 0.008), PAstV-3 (0.456 ± 0.01), and PAstV-4 (0.397 ± 0.014). High ENC values (>50) with a small amount of variation suggest a moderate bias but highly conserved codon usage pattern among the four subtypes of PAstVs. In summary, the overall and third-position nucleotide composition suggest that compositional constraints affect the codon usage pattern of PAstV genomes, and the PAstV CDSs were found to be rich in A/U nucleotides in comparison to G and C nucleotides.

### 3.3. RSCU Patterns of PAstV

**Figure 2:**
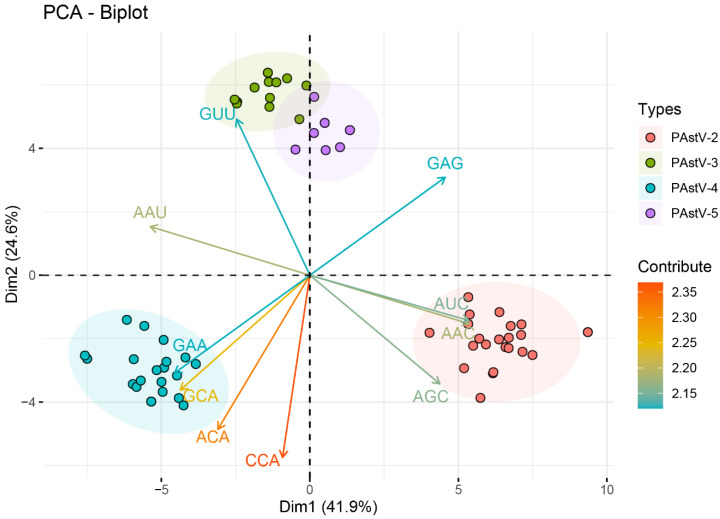
Principal component analysis (PCA) of the PAstV complete coding genomes. The first dimension was plotted against the second dimension. PAstV-2, PAstV-3, PAstV-4, and PAstV-5 are represented in orange, green, blue, and purple, respectively.

The codons of UGU[Cys] and GAU[Asp] were preferably used by all four PAstV subtypes. The preferred codon usage profiles of PAstV-3 and PAstV-5 were very similar: 16 out of 18 preferred codons were commonly used, with exceptions for the preferred codons of isoleucine and glutamine (Table 2). Among the 18 most abundantly used codons in the PAstV-2 genomes, 13 (GCC, GAG, UUC, GGC, CAC, AUC, AAG, CUC, AAC, CAG, CGC, GUC, and UAC) codons were C/G-ended, and the remaining 5 (UGU, GAU, CCA, UCA, and ACA) codons were A/U-ended. The numbers of A/U-ended preferred codons in the PAstV-3, PAstV-4 and PAstV-5 were 16, 16, and 14, respectively. From the RSCU analysis, PAstV-2 exhibited comparatively higher codon usage bias towards C/G-end, and PAstV-3, PAstV-4, and PAstV-5 showed relatively higher codon usage bias towards A/U-ended codons. The results of the preferred codons were consistent with those of the nucleotide composition analysis. Among 59 codons, only two preferred codons, CUC[Leu] and CCA[Pro], were overrepresented (RSCU values >1.6) in the PAstV-2 genomes, while the PAstV-3, PAstV-4, and PAstV-5 genomes contained five (CUU[Leu], CCU[Pro], CGU[Arg], UCU[Ser], and GUU[Val]), six (GCA[Ala], CUU[Leu], CCA[Pro], AGG[Arg], UCA[Ser], and ACA[Thr]), and six (GGU[Gly], CUU[Leu], CGU[Arg], UCU[Ser], ACU[Thr], and GUU[Val]) overrepresented (RSCU values > 1.6) codons, respectively. PCA results showed that the first two principal axes accounted for 41.9% and 24.6% of the total variation of RSCUs (Figure S1). The points representing the four subgroups of PAstV genomes were mapped and clustered in clearly separate regions, although a small degree of overlap existed between PAstV-3 and PAstV-5 (Figure 2). Taken together, the RSCU analyses revealed the RSCU patterns of four PAstV genotypes, and compositional constraints of third position nucleotides in codons (G/C-ending codons of PAstV-2 versus A/T-ending codons of PAstV-3, PAstV-4 and PAstV-5) had the most influence on the selection of the preferred codons. The trend of the 59 synonymous codon usages indicated that the evolution of the four genotypes of PAstVs might be influenced.

### 3.4. Dinucleotide Frequency Abundancy Influences the Codon Usage Bias of PAstV

**Figure 3:**
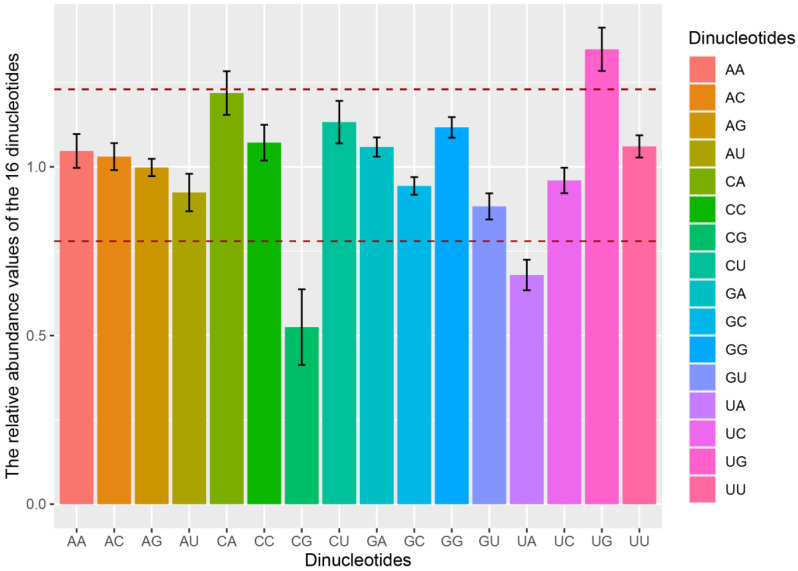
Dinucleotide abundancy of the complete CDS of PAstV. The different colors represent the different dinucleotides. Dinucleotides are regarded as underrepresented or overrepresented if the relative abundance values are below 0.78 or over 1.23 (dashed lines), respectively.

We performed a dinucleotide analysis on the four subtypes of PAstVs to understand the possible effect of dinucleotide frequencies on the codon usage. Dinucleotides UpG were overrepresented (*P_xy_* ≥ 1.23), whereas dinucleotides CpG and UpA were underrepresented (*P_xy_* ≤ 0.78) in the genome CDSs of the four subtypes of PAstVs (Table 3 and Figure 3). Additionally, dinucleotide CpA was overrepresented (*P_xy_* ≥ 1.23) in the genome CDSs of PAstV-2 and PAstV-4, and dinucleotide CpU was over-represented in the genome CDSs of PAstV-5. These results showed that significant biases of the dinucleotide content variation were observed in the four subtypes of PAstVs.

In order to determine the effect of dinucleotide usage on codon usage bias, we compared the over-representative and under-representative dinucleotides with preferred and under-representative codons. Among eight CpG-containing codons, five codons (GCG, CCG, CGA, UCG, and ACG) were under-represented (RSCU value < 0.6), indicating that dinucleotide CpG were inhibited. Furthermore, the RSCU values of all six UpA-containing codons (AUA, CUA, UUA, GUA, UAC, and UAU) were <1.6, suggesting that dinucleotide UpA were inhibited. Among all five UpG dinucleotides-containing codons, UGU codon was found to be a preferred codon, and RSCU values of all UpG dinucleotides-containing codons were >0.6. For all eight CpA dinucleotides-containing codons, five (CAC, CCA, CAG, UCA, and ACA) and six (GCA, CAU, CCA, CAA, UCA, and ACA) codons were preferentially used synonymous codons in PAstV-2 and PAstV-4, respectively. Of the eight CpU dinucleotides-containing codons, five codons (GCU, CUU, CCU, UCU, and ACU) were preferentially used in PAstV-2. These results indicated that dinucleotide abundance influences the codon usage bias of the four subtypes of PAstVs.

### 3.5. Identification of the Forces Influencing Codon Usage Patterns

**Figure 4:**
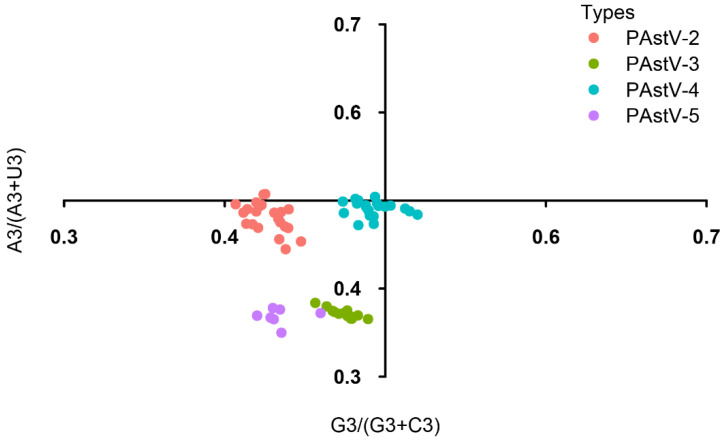
PR2 plot analysis of the PAstV complete coding genomes. PAstV-2, PAstV-3, PAstV-4, and PAstV-5 strains are represented in orange, green, blue, and purple, respectively.

**Figure 5:**
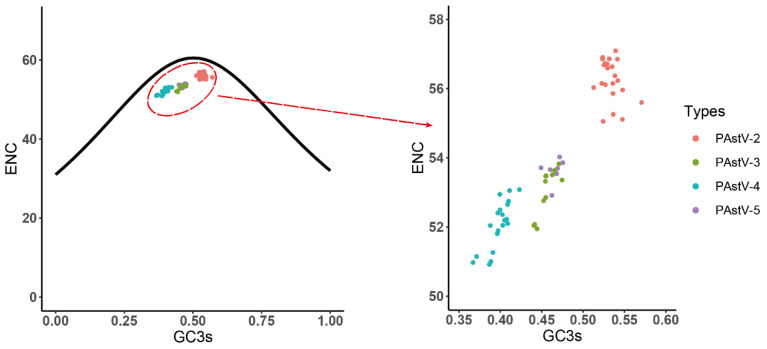
ENC plot analysis of the complete coding genomes of PAstV. The ENC plot displayed the relationships between ENC and GC content at the third codon position (GC3s) of protein-coding sequences. The curve represents the expected ENC values for all GC3 compositions. PAstV-2, PAstV-3, PAstV-4, and PAstV-5 strains are represented in orange, green, blue, and purple, respectively.

To evaluate the forces shaping the codon usage patterns in the four genotypes of PAstVs, PR2 bias, ENC plots, and neutrality analyses were carried out. In the PR2 bias analysis, significant deviations from the parity rules were observed (A ≠ U, C ≠ G) (Figure 4), indicating that the extent of the evolutionary forces shaping the codon usage patterns of the four subtypes of PAstVs were not unique. In the ENC plot, all of the ENC values of PAstV strains fell below but were close to the expected ENC curve (Figure 5). Additionally, sequences of PAstV-2 and PAstV-4 were clustered separately, whereas sequences of PAstV-3 and PAstV-5 were clustered together in the ENC plots. These results indicate that mutation pressure and natural selection led to the codon usage bias of the four genotypes of PAstVs.

**Figure 6:**
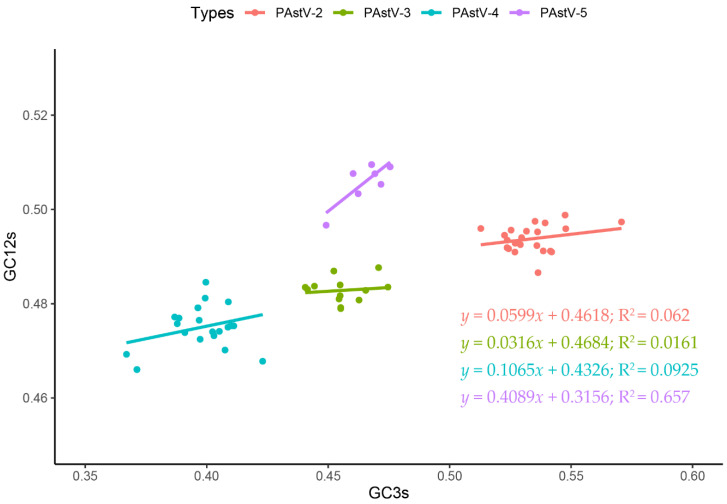
Neutrality analysis of the PAstV complete coding genomes. The neutrality plot displays the correlation between GC content at synonymous positions (GC12s) and GC content at non-synonymous positions (GC3s). PAstV-2, PAstV-3, PAstV-4, and PAstV-5 strains are represented in orange, green, blue, and purple, respectively.

The neutrality analysis between the GC3s and GC12s values was employed to determine the extent of the two evolutionary forces on the codon usage pattern of PAstV strains. A significant correlation between GC3s and GC12s was observed in the PAstV-5 strains (*y* = 0.4089*x* + 0.3156; R^2^ = 0.657; *p* < 0.01) (Figure 6). Thus, the percentage of constraints of natural selection was 59.11% for the PAstV-5 strains. No significant correlation between GC3s and GC12s was observed in the genomes of PAstV-2 (*p* = 0.2639; R^2^ = 0.062), PAstV-3 (*p* = 0.679; R^2^ = 0.0161), or PAstV-4 (*p* = 0.158; R^2^ = 0.0925) strains. Therefore, natural selection plays a dominant role in driving codon usage bias for these three subtypes. Overall, the above results indicate that the effect of directional mutation pressure is present in the codon usage of PAstV-5, but natural selection dominates the evolution of codon usage of the four subtypes of PAstVs.

### 3.6. PAstV Strains Adaptation to Host Species

**Figure 7:**
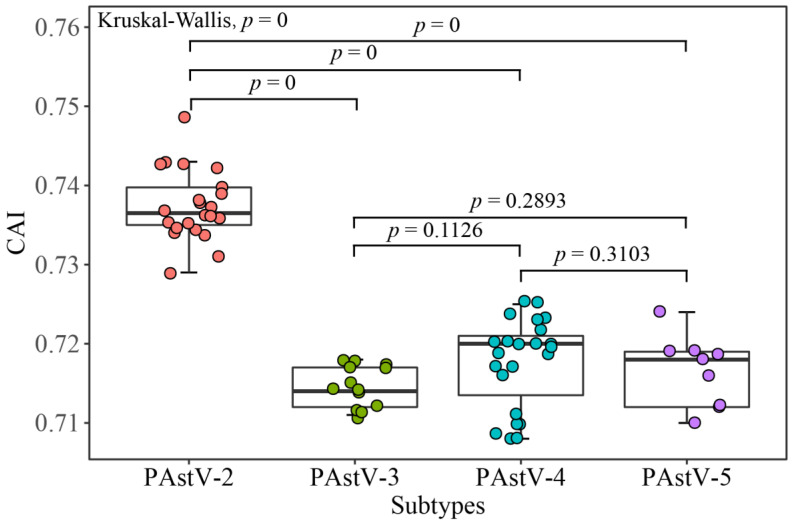
Codon adaptation index (CAI) of the PAstV complete coding genomes. PAstV-2, PAstV-3, PAstV-4, and PAstV-5 strains are represented in orange, green, blue, and purple, respectively.

**Figure 8:**
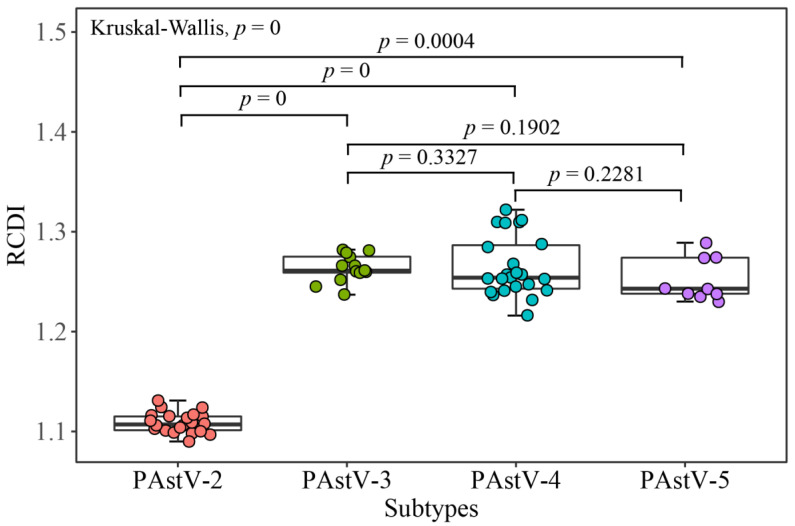
Relative codon deoptimization index (RCDI) of the PAstV complete coding genomes. PAstV-2, PAstV-3, PAstV-4, and PAstV-5 strains are represented in orange, green, blue, and purple, respectively.

**Figure 9:**
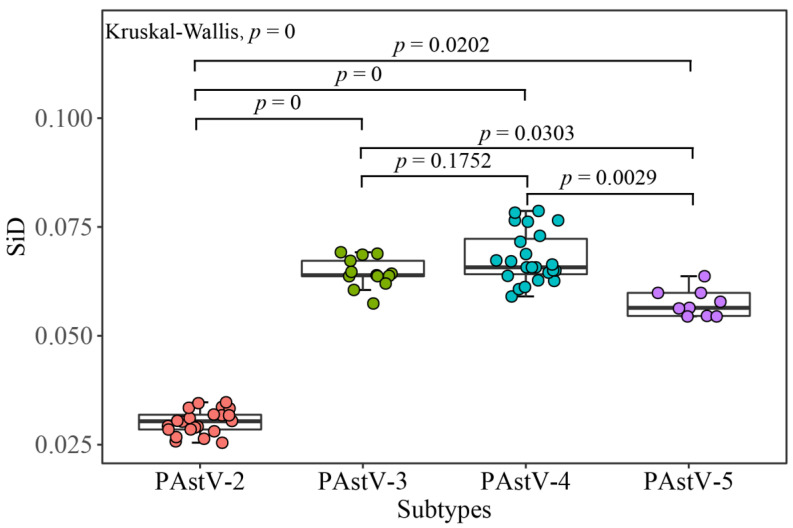
Similarity index (SiD) of the PAstV complete coding genomes. PAstV-2, PAstV-3, PAstV-4, and PAstV-5 strains are represented in orange, green, blue, and purple, respectively.

The analyses of CAI, RCDI, and SiD values were employed to evaluate the codon usage similarities between the PAstV strains and potential host species. The results based on CAI values show that PAstV presented the highest CAI value to ducks, followed by chickens, humans, dogs, horses, mice, pigs, cats, cattle, and sheep, while it was comparatively unsuitable for growth in the goat (Figure S2). PAstV-2 displayed the significant higher CAI values to pig compared with the other three subtypes of PAstVs (Figure 7). Comparable RCDI analysis showed that the mean RCDI values of PAstV-3, PAstV-4, PAstV-5 were significantly higher than PAstV-2 (Figure 8), suggesting the codon deoptimization of PAstV-2 is less than PAstV-3, PAstV-4, and PAstV-5. PAstV-3 and PAstV-4 were significantly higher than the PAstV-2 and PAstV-5 in SiDs (Figure 9), indicating that the pigs had a significantly deeper effect on PAstV-3 and PAstV-4 than PAstV-2 and PAstV-5.

## 4. Discussion

In this study, we analyzed the phylogenetic relationship of PAstV. Phylogenetic analysis demonstrated that the PAstV-5 occupied the basal position in the phylogenetic tree, indicating the multiple differentiations of PAstV. Given that the phylogenetic differentiation in porcine astrovirus might imply its evolutionary history, the identification of the phylogeny of porcine astrovirus provides valuable insight into the origin and evolution of porcine astrovirus.

To adapt to changes in the host and the environment, RNA viruses evolve by altering the composition of their genomes [42]. As an important indicator of viral evolution, codon usage preference is affected by many factors, including natural selection, mutation pressure, composition of the genomes or genome regions, and gene length [43]. To dissect evolutionary forces of codon usage bias, a total of 67 complete coding sequences of PAstV genomes were used to perform a comprehensive analysis of codon usage among PAstV-2, PAstV-3, PAstV-4, and PAstV-5.

The genotype-specific preferences of the four subtypes of PAstVs were observed in the third nucleotide position of the codons. More specifically, PAstV-2 tends to use the G/C ending codons, whereas PAstV-3, PAstV-4, and PAst-5 prefer A/U ending codons. Codon usage bias, largely determined by the nucleotide at the third position of the codon, allows a different perspective on the evolution of the virus [29]. Differences in the nucleotide usage of genome CDSs demonstrate that nucleotide composition indeed affects codon usage bias of the four subtypes of PAstVs.

The effective number of codons (ENC) was calculated to identify bias in the use of synonymous codons. High ENC values (>40) have been identified in many animal viruses, such as porcine circovirus 3 [44], porcine deltacoronavirus [45], and rabies virus [46]. In our study, the mean ENC values of PAstV-2, PAstV-3, PAstV-4, and PAstV-5 were 56.265 ± 0.602, 53.059 ± 0.656, 52.007 ± 0.678, and 53.647 ± 0.316, respectively, demonstrating that a low codon preference was present in the four subtypes of PAstVs. As suggested by previous reports [47,48], the four subtypes of PAstVs with low codon bias may have a selective advantage for their efficient replication in pigs.

In order to understand the codon usage patterns, RSCU values of 59 synonymous codons were estimated. The results of the RSCU analysis revealed that A/U-ended codons were preferentially used over G/C-ended codons in the genomes of PAstVs-3,4,5, while PAstV-2 tended to use G/C-ending preferred codons. The PCA plot showed a clear separation among different PAstV subtypes, indicating that synonymous codon usage is distinct for each subtype of PAstV strains. These results showed that despite being a single-stranded RNA virus with a very high mutation rate, PAstV has a relatively stable synonymous codon usage at a subtype level.

Although RSCU analysis is generally used to investigate synonymous codon usage patterns, it has limitations in revealing the forces that affect codon usage [49]. Therefore, the codon usage analysis was further carried out on the dinucleotides of the four subtypes of PAstVs. The results indicated remarkable divergence of dinucleotide patterns among the four subtypes of PAstVs. In coding sequences of PAstV genomes, dinucleotides CpG and UpA were underrepresented, and dinucleotide UpG was overrepresented. Dinucleotide CpA was specifically overrepresented in the genome CDSs of PAstV-2 and PAstV-4. The frequency of dinucleotides is affected by codon usage, mutation pressure, and natural selection [50]. CpA and UpG increases are regarded as a compensatory mechanism for both CpG and UpA reduction [51,52]. Low CpG content in viruses is usually considered to function to evade host defense and to be affected by natural selection [50,53]. UpA is another dinucleotide that is commonly underrepresented in viral genomes due to natural selection [48]. Decreasing the content of UpA can reduce the sensitivity of ribonuclease, which is conducive to maintaining the stability of mRNA [50] and to avoiding energy instability [54]. The results demonstrated that CpG and UpA were underrepresented in the four subtypes of PAstVs, suggesting that natural selection may have an important role in modeling the codon usage patterns of PAstV strains.

To better understand the roles of mutation pressure and natural selection in shaping the codon usage, PR2 analysis, ENC-GC3 plots, and neutrality analysis were performed. We found a non-proportional distribution from the parity rules, suggesting that both mutation pressure and natural selection contributed to codon usage bias of the four subtypes of PAstVs. The ENC-GC3 plot showed that the points representing PAstV sequences fell below the expected ENC curve. For the ENC-GC3s correlation analysis, if the codons were only affected by the mutation pressure, the actual ENC observations would fall above the ENC expectation curve on the plot of ENC against GC3s [55]. Conversely, if the actual observations of ENC values fall far below the expected curve of ENC values, it means that natural selection has played a major role in codon usage patterns [50]. Therefore, the analysis using the ENC plot indicated mutation pressure and natural selection has driven the codon usage bias of the four subtypes of PAstVs. Although the ENC-GC3s analysis provides a method for quantitative analysis of codon usage bias, this method does not accurately measure the contributions of natural selection and mutation pressure to the codon usage bias of a species [56,57]. To provide more information on this issue, neutral evolution analysis was performed. According to the neutrality plot, the codon usage patterns of PAstV subgroups were determined under different evolutionary pressures. Specifically, mutation pressure and natural selection contributed 40.89% and 59.11% to shaping codon usage pattern of PAstV-5. Natural selection accounted for 94.01%, 96.84%, and 89.35% driving the codon usage bias of PAstV-2, PAstV-3, and PAstV-4, respectively. Taken together, these results suggest that different evolutionary pressures are acting on the four subtypes of PAstVs. Both mutation pressure and natural selection influence codon usage patterns of PAstV-5, while natural selection is the dominant evolutionary force driving codon usage bias of PAstV-2, PAstV-3, and PAstV-4.

The emergence, dynamics, and evolution of viral diseases are determined by host-virus interactions. Intriguingly, all of the four subtypes of PAstVs have the highest CAI value to ducks among the 10 tested hosts. Multiple interspecies transmission events have occurred among human astroviruses, non-human mammalian astroviruses, and avian astroviruses [58]. There have been reports of *Avastrovirus* infecting mammalian species in ecotones, such as small and medium sized farms that rear multiple species [58,59]. The prevalent interspecies transmission of astroviruses reflects their varying origins. Codon usage study can easily predict the carrier hosts that may act as a source of infection in other co-circulating species [50]. The high CAI value of four subtypes of PAstVs to animals indicated a similar codon usage pattern between animals and PAstVs. We proposed the similar codon usage between PAstV and hosts might advance the cross-species transmission of astrovirus. The values of CAI, RCDI, and SiD may reveal the different adaptabilities of four subtypes of PAstVs to pigs. Of these, PAstV-2 may be most adaptive to pigs in theory than the others, in view of its high value of CAI and low values of RCDI and SiD. This might explain to some extent why PAstV-2 was found as the predominant genotype in many countries [60]. Future studies are warranted to pay more attention to the epidemiology and pathogenicity of PAstV-2 strains. 

Codon usage analysis could be used to design the protein-based vaccine against pathogenic viruses based on the control of viral protein expression. Attenuation by the deoptimizations of dinucleotides and/or codons has achieved as a rapid and efficient strategy for attenuation of various small RNA viruses which causes attenuation of viral virulence, and is used to the development of live, attenuated RNA virus vaccines with superior genetic stabilities [61,62,63,64]. Conversely, the optimizations of dinucleotides and/or codons in viral genes increase the protein expression level dramatically and are often performed for vaccine research to increase the immunogenicity of the target [64]. Besides, codon usage bias provides a theoretical basis for studying the transcript regulation, function, and pathological relevance of viral protein. A new transcription regulation was found in some persistent viruses which use poor codons in a distinctive way to temporarily regulate late expression of structural gene products [65]. Information regarding the codon usage pattern and host adaptability of the four subtypes of PAstVs may be useful to identify the potential hosts and the suitable experimental animal models for pathogenesis and vaccine researches.

## 5. Conclusions

To our knowledge, this study is for the first time to reveal the codon usage pattern for PAstVs. Phylogenetic analysis result showed the clade PAstV-5 occupies the basal position of the phylogenetic tree. The results from nucleotide composition analysis show that the genome CDSs of PAstVs-3,4,5 are rich in A/U nucleotides in comparison to G/C nucleotides. The C/G-ended codons are the preferentially used synonymous codons in the PAstV-2, whereas AU-ended codons were the preferred synonymous codons in the PAstV-3, PAstV-4, and PAstV-5. Natural selection and mutation pressure are the main factors influencing the codon usage bias in the PAstV-5 genome. The codon usages of PAstV-2, PAstV-3, and PAstV-4 are mainly constrained by selection pressure. The high similar codon usage between PAstV and animals might account for the broad host range and the cross-species transmission of astrovirus. Overall, the information from this study provides new insights for understanding PAstV evolution regarding codon usage pattern and host adaptability.

## Figures and Tables

**Table 1:** The nucleotide composition and properties of CDS of the PAstV genomes.

Catalogs	PAstV-2	PAstV-3	PAstV-4	PAstV-5	All
A	0.268 ± 0.004	0.247 ± 0.002	0.301 ± 0.003	0.238 ± 0.002	0.271 ± 0.024
C	0.263 ± 0.004	0.234 ± 0.003	0.222 ± 0.004	0.259 ± 0.005	0.243 ± 0.019
G	0.244 ± 0.003	0.24 ± 0.002	0.227 ± 0.003	0.234 ± 0.001	0.236 ± 0.008
U	0.225 ± 0.004	0.279 ± 0.002	0.25 ± 0.004	0.269 ± 0.004	0.25 ± 0.021
GC	0.507 ± 0.005	0.474 ± 0.004	0.449 ± 0.006	0.493 ± 0.005	0.479 ± 0.025
GC1s	0.549 ± 0.006	0.541 ± 0.003	0.528 ± 0.004	0.565 ± 0.007	0.543 ± 0.014
GC2s	0.438 ± 0.004	0.424 ± 0.003	0.422 ± 0.007	0.448 ± 0.003	0.431 ± 0.011
GC12s	0.494 ± 0.003	0.483 ± 0.003	0.475 ± 0.005	0.506 ± 0.004	0.487 ± 0.012
GC3s	0.534 ± 0.012	0.456 ± 0.01	0.397 ± 0.014	0.467 ± 0.008	0.463 ± 0.058
U3s	0.308 ± 0.014	0.433 ± 0.01	0.399 ± 0.011	0.421 ± 0.009	0.379 ± 0.052
C3s	0.371 ± 0.011	0.283 ± 0.008	0.241 ± 0.013	0.312 ± 0.011	0.302 ± 0.056
A3s	0.286 ± 0.011	0.256 ± 0.006	0.384 ± 0.01	0.245 ± 0.006	0.308 ± 0.057
G3s	0.276 ± 0.009	0.255 ± 0.008	0.235 ± 0.008	0.237 ± 0.005	0.253 ± 0.02
ENC	56.265 ± 0.602	53.059 ± 0.656	52.007 ± 0.678	53.647 ± 0.316	53.83 ± 1.902

**Table 2:** The relative synonymous codon usage (RSCU) of the CDS of PAstV strains. RSCU values of 59 synonymous codons excluding codons for Met and Trp are presented. The letter in brackets represents the abbreviation of the corresponding amino acid. The numbers of preferentially used synonymous codons are highlighted in green and orange colors. The RSCU values of the over-represented (RSCU > 1.6) codons are shaded using orange color.

Codon	PAstV-2	PAstV-3	PAstV-4	PAstV-5	All(G)
GCA(A)	1.129 ± 0.124	1.097 ± 0.084	1.813 ± 0.159	1.016 ± 0.05	1.343 ± 0.366
GCC(A)	1.331 ± 0.131	1.202 ± 0.074	0.874 ± 0.099	1.266 ± 0.103	1.14 ± 0.225
GCG(A)	0.568 ± 0.064	0.365 ± 0.061	0.34 ± 0.058	0.275 ± 0.049	0.411 ± 0.128
GCU(A)	0.972 ± 0.15	1.335 ± 0.09	0.972 ± 0.124	1.443 ± 0.082	1.106 ± 0.23
UGC(C)	0.98 ± 0.171	0.781 ± 0.187	0.71 ± 0.1	0.895 ± 0.18	0.837 ± 0.191
UGU(C)	1.02 ± 0.171	1.219 ± 0.187	1.29 ± 0.1	1.105 ± 0.18	1.163 ± 0.191
GAC(D)	0.961 ± 0.086	0.803 ± 0.06	0.692 ± 0.077	0.979 ± 0.102	0.84 ± 0.147
GAU(D)	1.039 ± 0.086	1.197 ± 0.06	1.308 ± 0.077	1.021 ± 0.102	1.16 ± 0.147
GAA(E)	0.834 ± 0.08	0.825 ± 0.08	1.163 ± 0.077	0.884 ± 0.075	0.952 ± 0.172
GAG(E)	1.166 ± 0.08	1.175 ± 0.08	0.837 ± 0.077	1.116 ± 0.075	1.048 ± 0.172
UUC(F)	1.072 ± 0.096	0.679 ± 0.055	0.702 ± 0.099	0.874 ± 0.121	0.842 ± 0.196
UUU(F)	0.928 ± 0.096	1.321 ± 0.055	1.298 ± 0.099	1.126 ± 0.121	1.158 ± 0.196
GGA(G)	0.791 ± 0.118	0.531 ± 0.081	1.055 ± 0.092	0.486 ± 0.086	0.79 ± 0.244
GGC(G)	1.189 ± 0.117	1.332 ± 0.119	0.896 ± 0.119	1.092 ± 0.065	1.103 ± 0.2
GGG(G)	0.88 ± 0.138	0.629 ± 0.065	0.729 ± 0.079	0.624 ± 0.099	0.745 ± 0.144
GGU(G)	1.14 ± 0.146	1.508 ± 0.069	1.32 ± 0.124	1.798 ± 0.1	1.362 ± 0.247
CAC(H)	1.183 ± 0.127	0.898 ± 0.105	0.688 ± 0.087	0.775 ± 0.068	0.903 ± 0.234
CAU(H)	0.817 ± 0.127	1.102 ± 0.105	1.312 ± 0.087	1.225 ± 0.068	1.097 ± 0.234
AUA(I)	0.738 ± 0.109	0.652 ± 0.1	0.783 ± 0.11	0.889 ± 0.028	0.757 ± 0.121
AUC(I)	1.356 ± 0.107	0.93 ± 0.048	0.916 ± 0.132	1.07 ± 0.034	1.084 ± 0.222
AUU(I)	0.906 ± 0.116	1.418 ± 0.097	1.301 ± 0.105	1.041 ± 0.033	1.159 ± 0.231
AAA(K)	0.927 ± 0.093	0.86 ± 0.037	0.989 ± 0.08	0.954 ± 0.036	0.939 ± 0.087
AAG(K)	1.073 ± 0.093	1.14 ± 0.037	1.011 ± 0.08	1.046 ± 0.036	1.061 ± 0.087
CUA(L)	0.635 ± 0.163	0.501 ± 0.079	0.561 ± 0.128	0.425 ± 0.067	0.555 ± 0.144
CUC(L)	1.639 ± 0.161	0.849 ± 0.062	1.127 ± 0.141	1.361 ± 0.132	1.273 ± 0.326
CUG(L)	0.953 ± 0.17	0.668 ± 0.062	0.699 ± 0.083	0.83 ± 0.089	0.794 ± 0.168
CUU(L)	1.582 ± 0.155	1.915 ± 0.093	1.886 ± 0.156	1.814 ± 0.215	1.782 ± 0.209
UUA(L)	0.404 ± 0.093	0.889 ± 0.127	0.715 ± 0.09	0.458 ± 0.121	0.612 ± 0.216
UUG(L)	0.787 ± 0.141	1.178 ± 0.138	1.011 ± 0.137	1.112 ± 0.105	0.983 ± 0.2
AAC(N)	1.213 ± 0.107	0.819 ± 0.09	0.774 ± 0.102	0.868 ± 0.063	0.939 ± 0.217
AAU(N)	0.787 ± 0.107	1.181 ± 0.09	1.226 ± 0.102	1.132 ± 0.063	1.061 ± 0.217
CCA(P)	1.802 ± 0.115	1.084 ± 0.07	2.029 ± 0.111	1.271 ± 0.066	1.669 ± 0.387
CCC(P)	0.789 ± 0.102	0.876 ± 0.049	0.49 ± 0.112	0.903 ± 0.077	0.718 ± 0.195
CCG(P)	0.393 ± 0.099	0.308 ± 0.038	0.218 ± 0.053	0.296 ± 0.052	0.303 ± 0.1
CCU(P)	1.016 ± 0.114	1.733 ± 0.082	1.264 ± 0.139	1.531 ± 0.094	1.309 ± 0.29
CAA(Q)	0.836 ± 0.118	1.068 ± 0.087	1.048 ± 0.075	0.899 ± 0.098	0.962 ± 0.14
CAG(Q)	1.164 ± 0.118	0.932 ± 0.087	0.952 ± 0.075	1.101 ± 0.098	1.038 ± 0.14
AGA(R)	0.815 ± 0.183	0.555 ± 0.083	1.458 ± 0.166	0.726 ± 0.127	0.973 ± 0.395
AGG(R)	1.298 ± 0.119	1.43 ± 0.121	1.865 ± 0.209	1.183 ± 0.061	1.503 ± 0.311
CGA(R)	0.479 ± 0.119	0.375 ± 0.079	0.401 ± 0.118	0.354 ± 0.047	0.415 ± 0.113
CGC(R)	1.486 ± 0.225	1.292 ± 0.14	0.803 ± 0.136	1.478 ± 0.152	1.213 ± 0.35
CGG(R)	0.852 ± 0.156	0.609 ± 0.078	0.425 ± 0.091	0.585 ± 0.093	0.622 ± 0.21
CGU(R)	1.07 ± 0.174	1.739 ± 0.142	1.048 ± 0.188	1.674 ± 0.169	1.274 ± 0.353
AGC(S)	1.005 ± 0.136	0.222 ± 0.044	0.465 ± 0.125	0.386 ± 0.058	0.584 ± 0.327
AGU(S)	0.819 ± 0.16	0.313 ± 0.069	1.008 ± 0.11	0.651 ± 0.105	0.763 ± 0.279
UCA(S)	1.442 ± 0.227	1.549 ± 0.147	2.068 ± 0.238	1.5 ± 0.097	1.685 ± 0.345
UCC(S)	1.184 ± 0.2	1.404 ± 0.181	0.755 ± 0.158	1.157 ± 0.098	1.076 ± 0.3
UCG(S)	0.48 ± 0.145	0.511 ± 0.14	0.225 ± 0.065	0.434 ± 0.099	0.392 ± 0.168
UCU(S)	1.069 ± 0.194	2.001 ± 0.171	1.48 ± 0.231	1.873 ± 0.131	1.499 ± 0.409
ACA(U)	1.347 ± 0.194	0.995 ± 0.091	2.023 ± 0.133	0.854 ± 0.048	1.445 ± 0.476
ACC(U)	1.314 ± 0.157	1.099 ± 0.096	0.717 ± 0.094	1.266 ± 0.095	1.061 ± 0.287
ACG(U)	0.39 ± 0.099	0.348 ± 0.087	0.182 ± 0.053	0.279 ± 0.066	0.296 ± 0.118
ACU(U)	0.949 ± 0.143	1.559 ± 0.096	1.078 ± 0.106	1.601 ± 0.111	1.199 ± 0.295
GUA(V)	0.418 ± 0.113	0.406 ± 0.044	0.713 ± 0.141	0.241 ± 0.038	0.493 ± 0.2
GUC(V)	1.309 ± 0.162	0.894 ± 0.087	0.918 ± 0.139	1.079 ± 0.117	1.064 ± 0.226
GUG(V)	1.164 ± 0.117	0.936 ± 0.103	1.004 ± 0.133	0.834 ± 0.117	1.021 ± 0.164
GUU(V)	1.109 ± 0.16	1.764 ± 0.072	1.365 ± 0.144	1.847 ± 0.159	1.423 ± 0.317
UAC(Y)	1.238 ± 0.092	0.815 ± 0.127	0.775 ± 0.11	0.812 ± 0.038	0.94 ± 0.233
UAU(Y)	0.762 ± 0.092	1.185 ± 0.127	1.225 ± 0.11	1.188 ± 0.038	1.06 ± 0.233

**Table 3:** Relative dinucleotides frequencies of CDS of PAstV strains. The odds ratios of over-represented (≥1.23) and the under-represented (≤0.78) dinucleotides are highlighted using orange and blue, respectively.

	PAstV-2	PAstV-3	PAstV-4	PAstV-5	All(G)
AA	1.001 ± 0.033	1.121 ± 0.015	1.039 ± 0.021	1.076 ± 0.021	1.047 ± 0.05
AC	1.049 ± 0.022	1.025 ± 0.021	0.991 ± 0.02	1.092 ± 0.017	1.03 ± 0.04
AG	0.977 ± 0.025	1 ± 0.018	1.011 ± 0.02	1.014 ± 0.014	0.998 ± 0.026
AU	0.967 ± 0.025	0.872 ± 0.021	0.949 ± 0.019	0.83 ± 0.026	0.924 ± 0.055
CA	1.239 ± 0.023	1.137 ± 0.027	1.279 ± 0.022	1.135 ± 0.022	1.219 ± 0.065
CC	1.028 ± 0.022	1.124 ± 0.028	1.11 ± 0.021	1.003 ± 0.021	1.072 ± 0.053
CG	0.642 ± 0.027	0.528 ± 0.023	0.384 ± 0.015	0.595 ± 0.01	0.525 ± 0.112
CU	1.072 ± 0.03	1.181 ± 0.02	1.126 ± 0.041	1.23 ± 0.02	1.133 ± 0.063
GA	1.055 ± 0.024	1.032 ± 0.023	1.062 ± 0.021	1.1 ± 0.011	1.059 ± 0.029
GC	0.928 ± 0.021	0.955 ± 0.023	0.954 ± 0.029	0.937 ± 0.017	0.943 ± 0.026
GG	1.09 ± 0.026	1.142 ± 0.009	1.129 ± 0.025	1.118 ± 0.026	1.117 ± 0.031
GU	0.92 ± 0.03	0.886 ± 0.022	0.851 ± 0.029	0.871 ± 0.016	0.883 ± 0.039
UA	0.659 ± 0.021	0.748 ± 0.023	0.647 ± 0.022	0.713 ± 0.02	0.679 ± 0.045
UC	0.987 ± 0.032	0.913 ± 0.014	0.956 ± 0.028	0.971 ± 0.027	0.96 ± 0.038
UG	1.349 ± 0.031	1.274 ± 0.02	1.418 ± 0.025	1.277 ± 0.018	1.349 ± 0.064
UU	1.043 ± 0.028	1.061 ± 0.019	1.085 ± 0.031	1.041 ± 0.024	1.061 ± 0.033

## Supplementary Materials

The following are available online at https://www.mdpi.com/1999-4915/12/9/991/s1, Figure S1: Scree plot of percentage of explained variances for each principal component of the RSCU values of PAstV complete CDS. Figure S2: CAI analysis of the PAstV complete coding genomes in relation to potential host species. Table S1: The detailed information of PAstV strains in NCBI nucleic acid database. Table S2: The nucleotide composition and properties of complete CDS of the PAstVs.
