# Supplementary material for: Comprehensive Analysis of Codon Usage on Porcine Astrovirus
Source: Viruses. 2020 Sep 6;12(9):991. doi: 10.3390/v12090991 (PMC7552017; doi:10.3390/v12090991)
Supplement: Supplementary file 1 [file viruses-12-00991-s001.zip › Supplementary_Files/PAstV_Supplementary_Figures.docx]

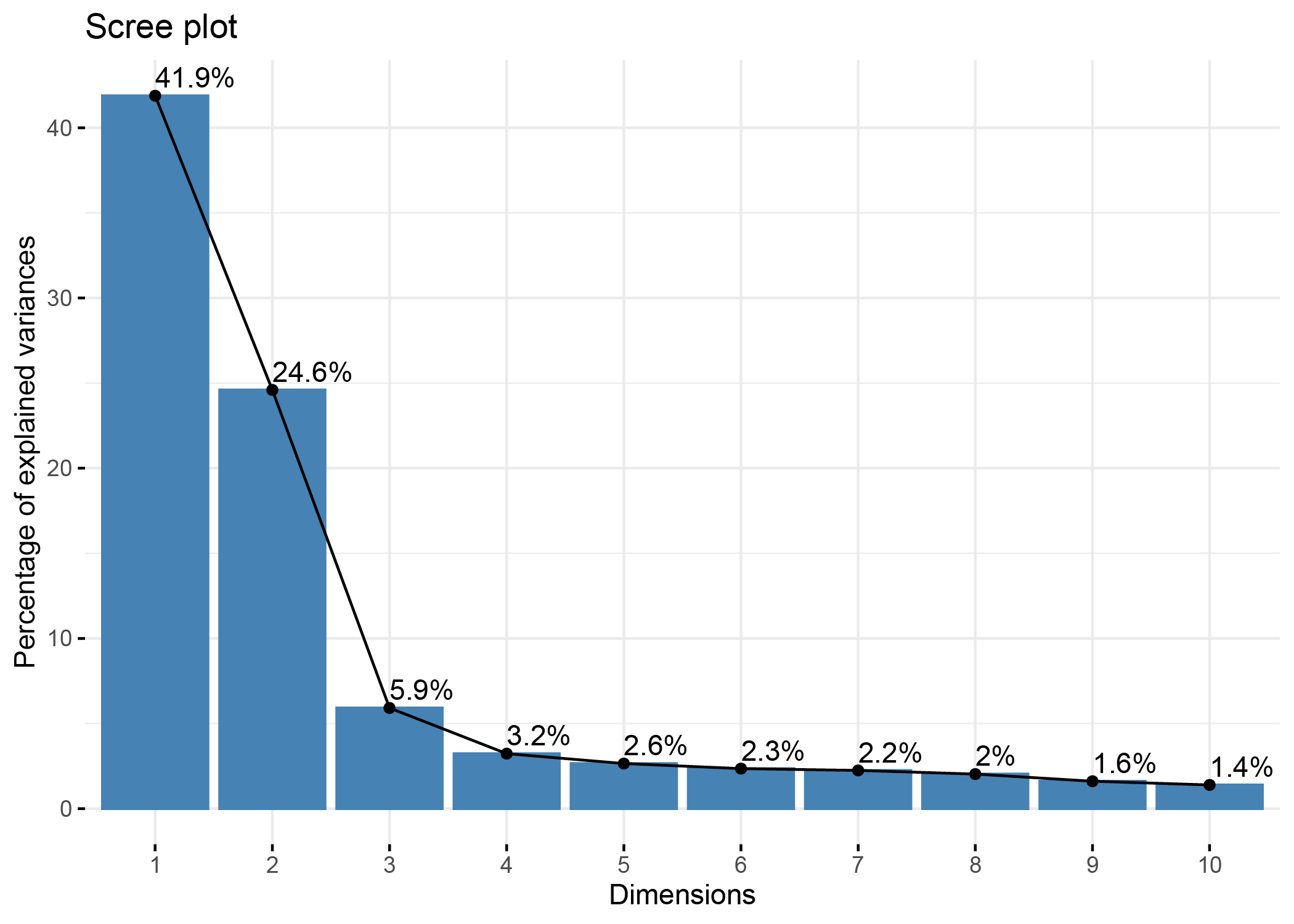


**Figure S1.** Scree plot of percentage of explained variances for each principal component of the RSCU values of PAstV complete CDS. This plot shows the proportion of variance in the RSCU values for each principal component (dimension), in descending order of magnitude. The scree plot confirms the first two dimension cumulate 66.5% variances.


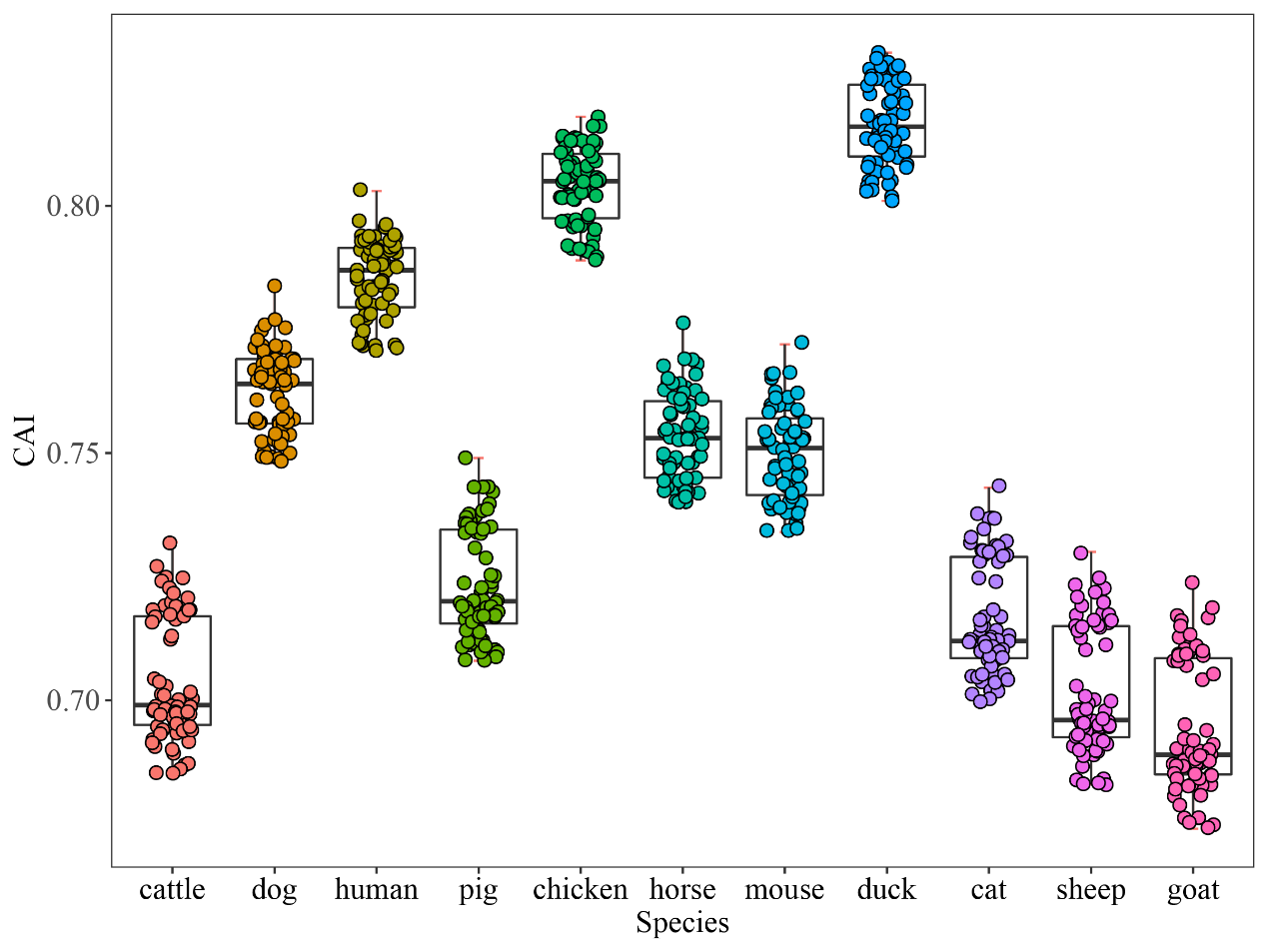


**Figure S2.** CAI analysis of the PAstV complete coding genomes in relation to potential host species.
